# 

**Published:** 1871-10

**Authors:** 


### BOOK NOTICES.

"The Eye, in Health and Disease," published by Alexander Moore, Boston; written by B. JOY JEFFRIES, A. M., M. D. 112 pages, 8vo, with thirty illustrations, $1.50. Anatomy and Physiology of the eye; spectacles, cataracts, squinting, injuries, and diseases; general care and treatment. 2 Hamilton Place, Boston. A valuable, reliable, and practical book.

"The Young Deliverers," by Rev. ELIJAH KELLOGG, published by Lee & Shepard. Also, "Married for Both Worlds," by same. Written by Mrs. A. E. PORTER.

"School-houses;" by JAMES JOHONNOT; "Architectural Designs," by S. E. HAWES; published by J. W. Schermerhorn & Co., New York, 8vo, 300 pages; dedicated to the Hon. Edward Cornell, founder of Cornell University, at Ithaca, New York. This is a most important, practical book; every teacher in the land, every school commissioner, and trustee, and superintendent, will fail of his duty to the public and to the rising generation, who does not at once purchase this very valuable book; it is full of wise, judicious, and practical suggestions, in reference to school-houses, teachers, etc., etc.

"Bible Sketches," for young people, by SAMUEL G. GREEN. By the American Tract Society, 150 Nassau Street, New York. An admirable volume to be placed in the hands of the young.

"Curiosities of the Law Reporters," by FRANKLIN FISKE HEARD, published by Lee & Shepard, Boston. A 12mo of 212 pages; a collection of Law Anecdotes, full of amusement and profitable suggestions withal.

"Thoughts for the Young Men and Women of America;" or a few practical words of advice to those born in poverty and destined to be reared in orphanage. By L. U. Reavis, St. Louis, Mo., and published by Samuel R. Wells, 389 Broadway, New York. It is one of the many useful and practical books issued by Mr. Wells, and is admirably adapted to instruct and encourage every poor youth. $1.00.

"New York Daily Witness," one cent each, of which the Editor, Publisher, and Proprietor, says: —

My aim is to introduce a pure and elevating Newspaper into every street, lane, and alley of this great city, and for its attainment I rely on four things:

1. And over all, the blessing of God.
2. The coöperation of His people.
3. The low price at which the paper will be sold in the streets, namely, one cent.
4. The variety and interest of its contents.

Nearly all Daily Newspapers are compiled in utter forgetfulness that three fourths of the population are women and children. They are intended generally for men, and only certain classes of them.

Weekly religious papers are exceedingly valuable, but they do not reach the masses. They are read by church-going people with great improvement, but they are unknown to those who need them most. To give the cream of them, therefore, in a cheap Daily to the masses, would be the supplying of a missing link of unspeakable importance between the Church and the world. John Dougal, 162 Nassau Street, New York.

"Aunt Dinah's Pledge." This is the title of a new book written by Miss Mary Dwinell Chellis, and published by the National Temperance Society. The great popularity of her former writings, "Out of the Fire," "Temperance Doctor," "Deacon Sim's Prayers," etc., will insure the new book a cordial welcome. It has been pronounced her best work by those who have read the advance sheets. Aunt Dinah was an eminent Christian woman. Her pledge included swearing and smoking, as well as drinking. It saved her boys, who lived useful lives and died happy, and by quiet, yet loving and persistent work, names of many others were added, who seemed almost beyond hope of salvation.

"Atlantic Monthly," $4.00 a year, Boston, Mass., has among other things a description of the noblest of Animals to wit —

### A PERFECT HORSE.

In weight she might have turned, when well conditioned, nine hundred and fifty pounds. In color she was a dark chestnut, with a velvety depth and soft look. About the hair indescribably rich and elegant. Many a time have I heard ladies dispute the shade and hue of her plush-like coat as they ran their white, jeweled fingers through her silken hair. Her body was round in the barrel, and perfectly symmetrical. She was wide in the haunches, without projection of the hip-bones, upon which the shorter ribs seemed to lap. High in the withers as she was, the line of her back and neck perfectly curved, while her deep, oblique shoulders and long, thick fore-arm, ridgy with swelling sinews, suggesting the perfection of stride and power. Her knees across the pan were wide, the cannon bone below them short and thin; the pasterns long and sloping; her hoofs round, dark, shiny, and well set on. Her mane was a shade darker than her coat, fine and thin, as a thoroughbred's always is whose blood is without taint or cross. Her ear was thin, sharply pointed, delicately curved, nearly black around the borders, and as tremulous as the leaves of an aspen. Her neck rose from the withers to the head in perfect curvature, hard, devoid of fat, and well cut up under the chops. Her nostrils were full, very full, and thin almost as parchment. The eyes, from which tears might fall or fire flash, were well brought out, soft as a gazelle's, almost human in their intelligence, while over the small bony head, over neck and shoulders — yea, over the whole body, and clean down to the hoofs, the veins stood out as if the skin were but tissue paper against which the warm blood pressed, and which it might at any moment burst asunder.

